# Effect of Pulsed Electric Fields (PEF) on Extraction Yield and Stability of Oil Obtained from Dry Pecan Nuts (*Carya illinoinensis* (Wangenh. K. Koch))

**DOI:** 10.3390/foods10071541

**Published:** 2021-07-03

**Authors:** Lourdes Melisa Rábago-Panduro, Mariana Morales-de la Peña, María Paz Romero-Fabregat, Olga Martín-Belloso, Jorge Welti-Chanes

**Affiliations:** 1 FEMSA Biotechnology Center, Tecnológico de Monterrey, School of Engineering and Sciences, Eugenio Garza Sada Ave. 2501, 64849 Monterrey, Mexico; a00821713@itesm.mx (L.M.R.-P.); olga.martin@udl.cat (O.M.-B.); 2 AGROTECNIO CERCA Center, Department of Food Technology, University of Lleida, Rovira Roure Ave. 191, 25198 Lleida, Spain; mariapaz.romero@udl.cat; 3 Bioengineering Center, Tecnológico de Monterrey, School of Engineering and Sciences, Epigmenio González Ave. 500, 76130 Queretaro, Mexico; mariana.moralesdlp@tec.mx

**Keywords:** pulsed electric fields, pecan nut oil, oil extraction yield, microstructural analysis, oil stability, enzyme activity

## Abstract

Pulsed electric fields (PEF) have been reported to increase the total oil extraction yield (OEY_TOTAL_) of fresh pecan nuts maintaining oil characteristics and increasing phenolic compounds in the remaining by-product. However, there is no information regarding the PEF effect on dry pecan nuts. Dry kernels were pretreated at three specific energy inputs (0.8, 7.8 and 15.0 kJ/kg) and compared against untreated kernels and kernels soaked at 3, 20 and 35 min. OEY_TOTAL_, kernels microstructure, oil stability (acidity, antioxidant capacity (AC), oil stability index, phytosterols and lipoxygenase activity), along with by-products phenolic compounds (total phenolics (TP), condensed tannins (CT)) and AC were evaluated. Untreated kernels yielded 88.7 ± 3.0%, whereas OEY_TOTAL_ of soaked and PEF-treated kernels were 76.5–83.0 and 79.8–85.0%, respectively. Kernels microstructural analysis evidenced that the 0.8 kJ/kg pretreatment induced oleosomes fusion, while no differences were observed in the stability of extracted oils. PEF applied at 0.8 kJ/kg also increased by-products CT by 27.0–43.5% and AC by 21.8–24.3% compared to soaked and untreated kernels. These results showed that PEF does not improve OEY_TOTAL_ when it is applied to dry pecan nuts, demonstrating that kernelsʹ moisture, oil content and microstructure play an important role in the effectiveness of PEF.

## 1. Introduction

Pulsed electric fields (PEF) involved the application of intermittent electric fields of varying intensity (0.1–50 kV/cm) and short duration (µs–ms) [1]. Currently, this technology has been used as a pretreatment to oil extraction from rapeseeds, sunflower seeds, sesame seeds and olives enhancing oil extraction yield (OEY) and increasing oil acidity [2,3,4,5]. The improvement of OEY has been attributed to the electroporation phenomenon, while the increase of oil acidity was associated with triacylglycerols hydrolysis by lipase activity. In a previous study, PEF were applied to fresh pecan nut kernels as a pretreatment employing different specific energy inputs [5]. Results indicated that PEF-treated kernels yielded 21.4% more oil than untreated kernels. Nonetheless, pecan nuts are usually submitted to a drying process for commercialization and the effect of this technology on the OEY of dry kernels has not been reported yet. Furthermore, studies concerning the PEF effect on seeds microstructure and oil acidity are scarce.

Pecan nut kernels (*Carya illinoinensis* (Wangenh. K. Koch)) are well-known for their high concentration of phenolic compounds, mono- and polyunsaturated fatty acids, phytosterols, and tocopherols [6,7]. Besides, pecan nut oil differentiates from other tree nut oils due to its content of polyunsaturated fatty acids (PUFA), β-sitosterol and γ-tocopherol [8,9]. Thus, to extend kernelsʹ shelf-life and avoid oil oxidation, a drying process is carried out reducing the moisture content (≤5.5%) and modifying kernels microstructure [10,11]. In pecan nuts, fatty acids are stored as triacylglycerols within oleosomes. These organelles are formed by a monolayer of phospholipids, proteins, and enzymes like lipoxygenase that is the key enzyme in lipids oxidation [12,13,14,15].

During oxidative deterioration, lipids are oxidized to hydroperoxides and then to secondary oxidation products that increment free radicalsʹ concentration. Hence, these compounds are considered important biomarkers to measure oil oxidative deterioration [16]. Acidity is the measurement of free fatty acids concentration produced by ester bonds hydrolysis of lipids by either enzyme action, heat or moisture [17]. Thus, its increment is related with a loss of oil stability. Another method to evaluate oil stability is the oil stability index (OSI). In the OSI method, oil is oxidized by air and high temperatures, producing volatile acids that are dissolved in water to follow the change in electrical conductivity [15]. The OSI value expresses the time necessary to complete oil oxidation at given experimental conditions being indicated by a drop of conductivity. Hence, a high OSI value implies a high resistance to oxidation of the analyzed oil [17].

This study was carried out to investigate the effect of PEF on OEY, OEY_TOTAL_ and microstructure of dry pecan nuts. In addition, the stability of the extracted oil was evaluated by determination of acidity, antioxidant capacity, OSI, phytosterols concentration and LOX activity as well as phenolic compounds and antioxidant capacity of the generated by-products hereinafter referred to as cakes.

## 2. Materials and Methods

### 2.1. Chemicals

Acetonitrile, ethyl acetate, methanol (MeOH), hexane, water HPLC grade (H_2_O), β-mercaptoethanol (β-ME), 2,2-diphenyl-1-picrylhydrazyl (DPPH), boric acid (H_3_BO_3_), hydrochloric acid (HCl), glutaraldehyde, osmium tetroxide, polyvinylpolypyrrolidone (PVPP), potassium hydroxide solution (KOH 0.1 M), sodium acetate (CH_3_COONa), sodium phosphate monobasic (NaH_2_PO_4_), sodium phosphate dibasic (Na_2_HPO_4_), Triton X-114 and uranyl acetate were acquired from Sigma-Aldrich (St. Louis, MO, USA) along with 6-hydroxy-2,5,7,8-tetramethylchromane-2-carboxylic acid (Trolox), linoleic acid, β-sitosterol, stigmasterol, campesterol, catechin, gallic acid and vanillin. Epoxy EMbed 812 resin was purchased from Electron Microscopy Sciences (Hatfield, PA, USA). Acetic acid (CH_3_COOH), ethanol (EtOH), sodium carbonate (Na_2_CO_3_) and KOH were purchased from DEQ (Monterrey, Nuevo León, Mexico).

### 2.2. Pecan Nuts

Kernel halves of dry pecan nuts (*Carya illinoinensis*) from Alesto brand were purchased in a local market in Lleida (Spain). In this study, dry kernels were separated in three experimental groups comparing PEF-treated kernels against reference and control kernels. No soaking nor PEF processing was performed on reference kernels, while control kernels were placed in tap water (1:3 *w*/*w*) for 3, 20 and 35 min to assess the effect of soaking at each PEF pretreatment. Kernels pretreated by PEF were immersed in tap water and processed. After a 10 min draining, control and PEF-treated kernels (1.0 g) were separated for moisture and microstructural analysis. Before freeze-drying, kernel samples (2.0 g) were taken for determination of lipoxygenase (LOX) activity. The remaining kernels were frozen (−16 °C, 24 h), freeze-dried (−50 °C, 1 mbar, 72 h), and stored at −40 °C until oil extraction.

### 2.3. Pulsed Electric Fields Application

The application of PEF was performed in a batch-system using a 20 × 8 cm methacrylate container with stainless-steel parallel electrodes as the treatment chamber. The batch-system was equipped with a 0.1 µF capacitator (Physics International, USA), a pulse generator (PT-55, Pacific Atlantic Electronics Inc., El Cerrito, CA, USA) and a TG-70 gas control unit. Based on a previous study [18], dry kernels were immersed in tap water (1:3 *w*/*w*) to apply 10, 99 and 192 monopolar exponential-wave pulses at an electric field strength of 5.0 kV/cm. These electrical conditions equaled to specific energy inputs of 0.8, 7.8 and 15.0 kJ per kg of pecan nut kernels in wet basis (*W*, kJ/kg wb), respectively.

### 2.4. Oil Mechanical Extraction

Oil extraction was performed on freeze-dried kernels (85.0 g) employing a screw-type press (YD-ZY-02A, Yoda Europe, Hangzhou, Zhejiang, China). Pecan nut oil was stored in 50 mL centrifuge tubes flushing N_2_ into the headspace and the generated cakes were placed in polyethylene bags and vacuum sealed. Oil and cake samples were stored at −40 °C until analyses.

#### Oil Extraction Yield

Reference, control and PEF-treated kernels oil extraction yields (OEY, %) were calculated as follows:

(1) $$\mathrm{OEY}=\frac{\left(m_{K}\times L_{K}\right)-(m_{C}\times L_{C})}{\left(m_{K}\times L_{K}\right)}\times 100,$$

where $m_{K}$ and $L_{K}$ are kernels mass (g) and oil content (g/100 g), and $m_{C}$ and $L_{C}$ are cakes mass and oil content, all in dry basis (db). Next, the oil extracted into the soaking water ($o_{\mathrm{SW}}$) of control and PEF-treated kernels was determined using the oil content of reference kernels ($L_{\mathrm{Reference}}$):

(2) $$o_{\mathrm{SW}}=\left(m_{K}\times L_{\mathrm{Reference}}\right)-\left(m_{K}\times L_{K}\right),$$

Total oil extraction yield (OEY_TOTAL_, %) of control and PEF-treated kernels was estimated using the $o_{\mathrm{SW}}$:

(3) $${\mathrm{OEY}}_{\mathrm{TOTAL}}=\frac{\left[\left(m_{K}\times L_{K}\right)-(m_{C}\times L_{C}\right)]+o_{\mathrm{SW}}}{\left(m_{K}\times L_{K}\right)}\times 100,$$

### 2.5. Kernels Analysis

#### 2.5.1. Moisture

The AOAC Official Method 920.151 [19] was used to analyze kernels moisture. Results were expressed as g per 100 g of kernels db (g/100 g db).

#### 2.5.2. Oil Content

Oil content was analyzed as reported by Villarreal-Lozoya et al. [20]. Samples and hexane (1:10 *w*/*v*) were homogenized (6000 rpm, 1.5 min) using an IKA^®^ T25 ultraturrax (IKA, Staufen im Breisgau, Germany), centrifuged (8500 rpm, 15 min, 20 °C) and supernatants collected. This procedure was repeated three times. Hexane was evaporated from pooled supernatants (25 rpm, 45 °C) and oil content was determined gravimetrically following the AOAC Official Method 960.39 [19]. Oil content was expressed as g per 100 g of freeze-dried kernels and cakes db (g/100 g db).

#### 2.5.3. Microstructural Analysis

Control and PEF-treated kernels were fixed employing the procedure reported by Kendall et al. [21] with modifications. Glutaraldehyde and osmium tetroxide solutions were made using 0.1 M phosphate buffer (pH 7.2). Kernels were fixed in 2.5% glutaraldehyde solution and left overnight. Next, samples were washed three times in 0.1 M phosphate buffer and postfixed in 1.0% osmium tetroxide solution for 2 h. Subsequently, samples were washed twice with 0.1 M acetate buffer, incubated with 0.5% uranyl acetate for 30 min and rinsed two times in 0.1 M acetate buffer. Kernels were dehydrated in an acetonitrile series (30–100%) before embedding in epoxy EMbed 812 resin and polymerizing (48 h, 60 °C). A Reichert Jung Ultracut E microtome (Leica Microsystems, Washington, DC, USA) was used to obtain semithin and ultrathin sections. These sections were stained with Richardson blue [22] and examined at 20× and 100× by light microscopy in an Olympus BX41 microscope (Olympus, Allentown, PA, USA).

#### 2.5.4. Lipoxygenase Activity

Kernels enzymatic extracts were obtained as described by Christopoulos and Tsantili [23]. An extraction solution was prepared by dissolving β-ME (5.0 mM), PVPP (1:100 *w*/*v*) and Triton X-114 (0.05:100 *w*/*v*) in 50 mM phosphate buffer (pH 6.6). Kernels (2.0 g) and the extraction solution (10 mL) were homogenized (6000 rpm, 40 s), filtered using glass wool and centrifuged (8000 rpm, 15 min, 4 °C). Supernatants were collected for determination of lipoxygenase (LOX) activity according to the procedure reported by Li et al. [24]. Solutions were made using 0.2 M borate buffer (pH 9.0). Linoleic acid dissolved in EtOH and 0.2 M borate buffer (1:1:1000 *v*/*v*/*v*) was employed as substrate stock solution to measure LOX activity. The stock solution (5 mL) was diluted completely in 20 mL of 0.2 M borate buffer and 5 mL of distilled water. The diluted solution (2 mL) and 0.2 M borate buffer (950 µL) were pipetted in a cell quartz and mixed by inversion. Next, enzymatic extracts (50 µL) were added and mixed by inversion. Absorbance was measured at 234 nm and registered every 10 s until 3 min of reaction in a Cecil CE 1010 UV-VIS spectrophotometer (Cecil Instruments Ltd., Cambridge, UK). LOX activity was calculated employing the molar extinction coefficient (ε) of hydroperoxides (26,800/M·cm) to express it as µmol of hydroperoxide produced per L of LOX per s (µmol/L·s) [25].

### 2.6. Oil Analysis

#### 2.6.1. Acidity

Oil acidity was performed following the AOAC Official Method 940.28 [19] and results were expressed as mg KOH per 100 g of pecan nut oil (mg KOH/100 g).

#### 2.6.2. Antioxidant Capacity

The DPPH radical scavenging capacity method reported by Gao et al. [26] was used to evaluate oil antioxidant capacity (AC). Pecan nut oil (1 mL) was diluted in ethyl acetate (10 mL) and mixed with 2 mL of 0.50 mM DPPH solution. The mixture was vortexed and left to react in darkness for 15 min. After this time, absorbance was measured at 515 nm. A standard curve of Trolox was prepared (0.003–0.030 mg/mL) to express results as mg Trolox equivalents per 100 g of pecan nut oil (mg Trolox EQ/100 g).

#### 2.6.3. Oil Stability Index

The stability index (OSI) of extracted oils was measured in a Rancimat 679 apparatus (Metrohm AG, Herisau, Switzerland) [27]. Oil samples (3.0 g) were heated at 110 °C while air was bubbled at a flow rate of 20 L/h and volatile products were dissolved in deionized water (60 mL). OSI values were obtained by Metrodata Version 1.0 software (Metrohm AG, Switzerland) and expressed in hours (h).

#### 2.6.4. Phytosterols Concentration

Extraction and quantification of phytosterols were done according to Domínguez-Avila et al. and Nair et al., respectively [28,29]. HCl and KOH solutions were made using EtOH as solvent. Oil was mixed with 6 M HCl (1:5 *w*/*v*) and incubated (1 h, 80 °C). The mixture was cooled in a water bath and 5 mL of 1.3% KOH was added and left to react (30 min, 80 °C). To phytosterols extraction, 2 mL of distilled water and 5 mL of hexane were included to the mixture, vortexed for 1 min, and centrifuged (3750 rpm, 15 min, 20 °C). The addition of distilled water and hexane was performed twice. Hexane was evaporated from pooled supernatants using a vacuum evaporator (2.5 h, 45 °C). Extracts were reconstituted in 0.5 mL of hexane for chromatographic analysis. A HPLC-ELSD system (Agilent 1200, Agilent Technologies, Santa Clara, CA, USA) equipped with a 5 µm, 4.6 mm × 500 mm Luna C8 column (Torrance, CA, USA) was employed to identify and quantify phytosterols from pecan nut oil. Column and ELSD temperature were maintained at 40 °C and 50 °C, respectively. Aliquots (10 µL) were analyzed employing a mobile phase consisted of MeOH:H_2_O (95:5 *v*/*v*) at a flow rate of 1 mL/min and the detector was set at a gain of 16. Standard curves of β-sitosterol (0.2–1.2 mM), stigmasterol (0.2–1.2 mM), and campesterol (0.05–0.25 mM) were prepared to quantification. Concentrations were expressed as mg per kg of pecan nut oil (mg/kg).

### 2.7. Cakes Analysis

After the determination of cakes oil content, the defatted portion was used for phenolic compounds analysis. Total phenolics (TP), condensed tannins (CT), and AC of reference, control and PEF-treated cakes were performed following the procedures reported by Rábago-Panduro et al. [18]. Results were expressed as mmol equivalents per 100 g of defatted cake db: TP was expressed as mmol gallic acid EQ/100 g db, CT as mmol catechin EQ/100 g db and AC as mmol Trolox EQ/100 g db.

### 2.8. Statistical Analysis

PEF pretreatments, oil extraction and LOX activity were performed by triplicate while oil and cake analysis were done by duplicate to a total of six replicates per experimental group. One-way ANOVA and Dunnett test were performed with the Minitab^®^ Version 18.1 software (Minitab, USA).

## 3. Results

### 3.1. Pecan Nut Kernels

#### 3.1.1. Moisture and Oil Content

The moisture of dry pecan nuts used as reference was 2.4 ± 0.1 g/100 g db, while control kernels soaked at 3, 20 and 35 min had moisture contents of 13.5 ± 0.6, 21.1 ± 0.9, and 21.7 ± 0.7 g/100 g db, respectively. In PEF-treated kernels moisture contents also increased reaching values of 13.5 ± 1.0, 18.0 ± 0.1 and 19.3 ± 1.1 g/100 g db at 0.8, 7.8 and 15.0 kJ/kg pretreatments, respectively. However, the moisture of kernels processed at 7.8 and 15.0 kJ/kg was significantly lower than their respective control kernels (*p* = 0.004 and *p* = 0.015, respectively). Regarding the oil content, reference kernels contained 69.4 ± 0.7 g/100 g db, decreasing to 62.7 ± 0.0 g/100 g db in control kernels and 62.7 ± 0.7 g/100 g db in kernels pretreated by PEF, containing 9.7% less oil than reference kernels. Likewise, the $o_{\mathrm{SW}}$ was 5.7 ± 0.0 g for both control and PEF-treated kernels.

#### 3.1.2. OEY, OEY_TOTAL_ and Microstructure

**Figure 1:**
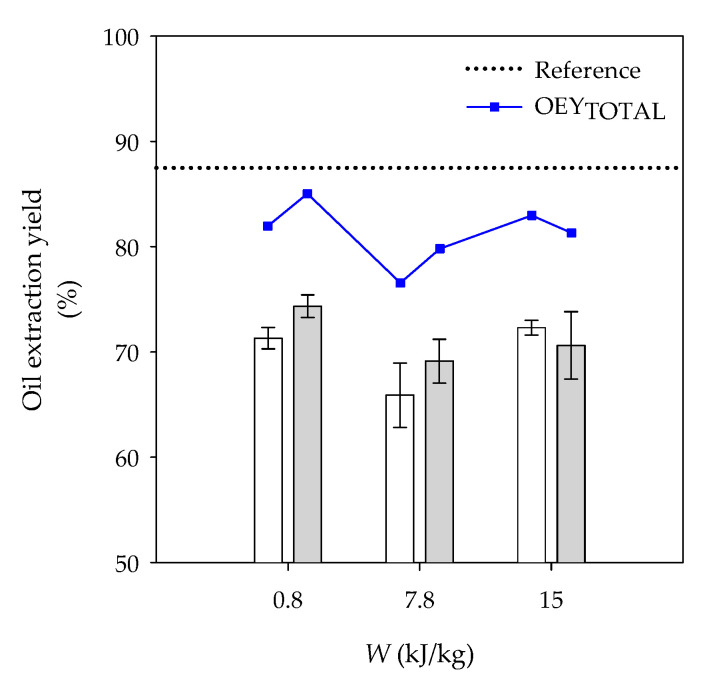
Oil extraction yields and total oil extraction yields (OEY_TOTAL_) of dry pecan nuts. Reference kernels were not soaked nor PEF processed. Control kernels (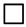 ) were placed in tap water for 3, 20 and 35 min corresponding to PEF-treated kernels (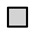) processed at 0.8, 7.8 and 15.0 kJ/kg, respectively. OEY_TOTAL_ is the oil extraction yield estimated with the $o_{\mathrm{SW}}$.

OEY and OEY_TOTAL_ of dry pecan nuts are displayed in Figure 1. The highest OEY was observed for reference kernels, being 88.7 ± 3.0%. Control kernels yielded 71.3 ± 1.0, 65.9 ± 3.1 and 72.3 ± 0.7% at 3, 20 and 35 min of soaking, respectively, while OEY of PEF-treated kernels was 74.3 ± 1.1, 69.1 ± 2.1 and 70.6 ± 3.2% at 0.8, 7.8 and 15.0 kJ/kg pretreatments, respectively. No statistical differences were observed between OEY of control and PEF-treated kernels submitted to comparable soaking times.

After considering the $o_{\mathrm{SW}}$, OEY_TOTAL_ of control kernels increased up to 82.0 ± 1.0, 76.5 ± 3.1 and 83.0 ± 0.7% for 3, 20 and 35 min of soaking, respectively, and OEY_TOTAL_ of PEF-treated kernels rose to 85.0 ± 1.1, 79.8 ± 2.1 and 81.3 ± 3.2% for 0.8, 7.8 and 15.0 kJ/kg pretreatments, respectively (Figure 1). No statistical differences were observed between control and PEF-treated kernels. Kernels processed at 0.8 kJ/kg were selected for the microstructural analysis.

**Figure 2:**
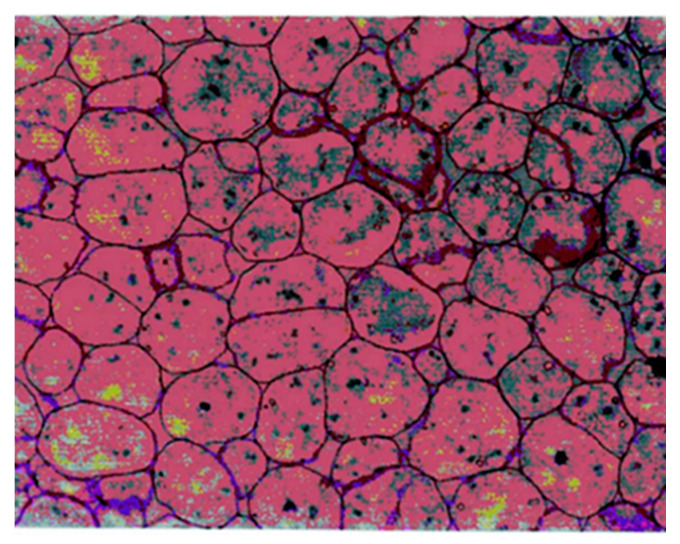
Light microscopy micrograph of cotyledon tissue of dry pecan nuts reported by Wakeling et al. [30] employed to compare the microstructure of control and PEF-treated kernels.

In order to compare the microstructure of PEF-treated and control kernels, a micrograph of the transversal section of dry kernels cotyledon tissue reported by Wakeling et al. was employed (Figure 2) [30]. In this micrograph, cells delimited by the cell wall containing intracellular oleosomes stained with toluidine blue were showed.

**Figure 3:**
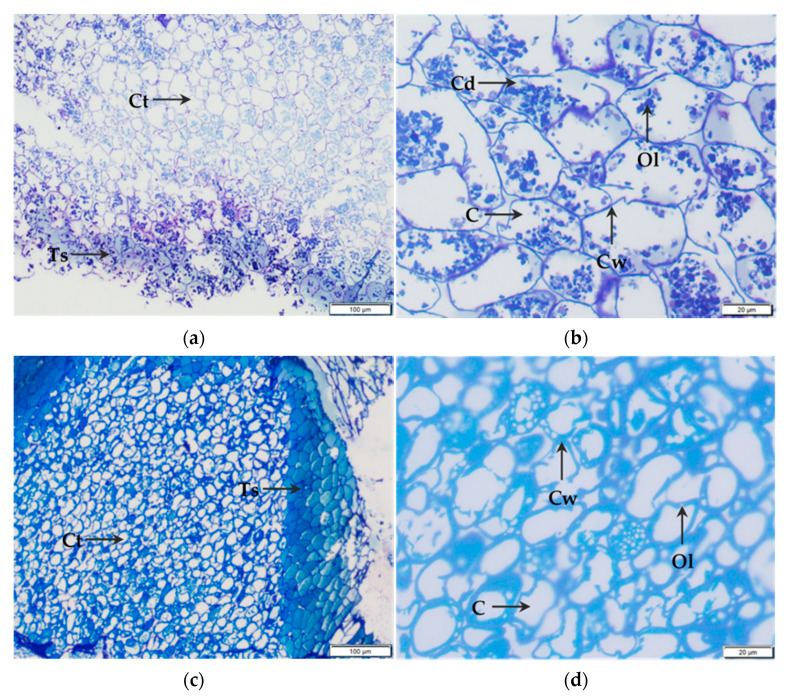
Light microscopy micrographs of testa and cotyledon tissue of dry pecan nuts. Control kernels (**a**,**b**) were placed in tap water for 3 min while PEF-treated kernels (**c**,**d**) were processed at 0.8 kJ/kg. Ts, testa; Ct, cotyledon tissue; C, cell; Cd, damaged cell; Cw, cell wall; Ol, oleosomes.

In the light microscopy of control kernels (Figure 3a), the cotyledon tissue exhibited similarities to the micrograph of Wakeling et al. [30] displaying delimited cells and oleosomes within the intracellular space stained with Richardson blue. However, at higher magnification, it appeared that the cotyledon tissue was composed of both intact and damaged cells. Additionally, oleosomes seemed to change their shape and aggregate within the intracellular space (Figure 3b). Concerning kernels pretreated by PEF, micrographs showed compaction of cells in testa and cotyledon tissues with a loss of delimited inclusions in the intracellular space (Figure 3c). It seems that PEF processing, rather than inducing the cell rupture, produced the rupture of intracellular inclusions. In Figure 3d, the higher magnification of PEF-treated kernels cotyledon tissue showed no difference between intact and damaged cells due to oleosomes fusion in the periphery of the cell.

### 3.2. Pecan Nut Oil Stability

The acidity of oils extracted from dry pecan nuts ranged from 19.0 ± 0.9 to 21.8 ± 0.9 mg KOH/100 g (Table 1). Only the oil extracted from kernels processed at 15.0 kJ/kg was significantly different, being 14.2% higher than its control. Concerning AC of extracted oils, it varied from 45.7 ± 2.2 to 49.0 ± 1.7 mg Trolox/100 g with no significant differences between experimental groups (Table 1).

Based on acidity and AC results, OSI, phytosterols concentration and LOX activity were determined for kernels pretreated at 0.8 kJ/kg, their control kernels, and reference kernels. The OSI values were 10.4 ± 0.4, 10.9 ± 0.3 and 10.6 ± 0.5 h for reference, control and PEF-treated oils, respectively. Neither soaking nor PEF processing significantly changed the stability index of pecan nut oil. Regarding the phytosterols content, oil from reference, control and PEF-treated kernels contained a β-sitosterol concentration of 929.0 ± 89.3, 858.2 ± 62.6 and 910.5 ± 132.2 mg/kg, respectively, while stigmasterol ranged from 324.4 ± 48.5 to 501.5 ± 79.7 mg/kg (Table 2). Campesterol was not detected despite that it has been reported in low concentration in pecan nuts [31,32]. Neither β-Sitosterol nor stigmasterol significantly changed among extracted oils.

The LOX activity of reference, control and PEF-treated kernels was 4.52 ± 0.14, 4.83 ± 0.10 and 4.41 ± 0.20 µmol/L·s, respectively. Reference and PEF-treated kernels showed LOX activity values significantly lower than control kernels (*p* = 0.006 and *p* = 0.001, respectively). It seemed that the application of PEF at 0.8 kJ/kg did not promote lipid oxidation of pecan nut oil. These results agreed with acidity, AC, and OSI values of the oil extracted from kernels processed at 0.8 kJ/kg. However, it should not be discarded lipid oxidation via enzyme activation since the acidity of pecan nut oil increased at higher *W*.

### 3.3. Pecan Nut Cakes

TP, CT and AC of cakes generated from dry pecan nuts are presented in Table 3. The reference cake showed TP, CT and AC values of 20.0 ± 1.0 mmol gallic acid EQ/100 g db, 15.4 ± 1.0 mmol catechin EQ/100 g db and 14.7 ± 1.0 mmol Trolox EQ/100 g db, respectively. TP varied from 18.1 ± 0.7 to 20.6 ± 1.8 mmol gallic acid EQ/100 g db in control cakes while, in PEF-treated cakes, TP decreased by 20.9% at 0.8 kJ/kg compared to its control (Table 3). The application of 0.8 and 7.8 kJ/kg increased the CT content of cakes by 27.0 and 10.7%, respectively, compared to their respective control cakes. An overall improvement in AC of cakes generated from PEF-treated kernels was observed with the highest increment observed at 0.8 kJ/kg, increasing by 24.3% in comparison with its control.

## 4. Discussion

Dry pecan nuts used as reference kernels displayed moisture and oil content within ranges reported in other studies [10]. Regarding kernelsʹ immersion in water, moisture reductions observed in kernels pretreated at 7.8 and 15.0 kJ/kg could be associated with the release of intracellular water as a result of electroporation of the cell membrane during PEF processing [33,34]. Oil content reductions along with $o_{\mathrm{SW}}$ values were comparable to those reported for fresh kernels pretreated by PEF [18], being related to the exposure of kernels cotyledon tissue to the soaking water provoking oleosomes release. An increment of oleosomes extraction was expected for dry kernels compared to the fresh ones as a result of the drying effect on kernelsʹ microstructure [11]. However, given that the $o_{\mathrm{SW}}$ was comparable among pecan nuts, it is proposed that kernels rehydration and water-soluble compounds release were favored over oleosomes extraction.

Concerning oil extraction from dry pecan nuts, the OEY of reference kernels (88.7%) was higher than those reported for pecan nut oil extracted by hydraulic pressing (56.4–58.9%) [9,35,36] and higher than the OEY of fresh pecan nut kernels used as a reference in the previous study (63.8%) [18]. Dry kernels differed from fresh kernels in moisture and oil content but also in kernels microstructure displaying more structural damage compared to fresh pecan nuts [37,38,39]. Savoire et al. evaluated oil extraction processes comparing seeds from the same type that only differed in moisture and oil content. The authors reported an overall improvement of OEY in seeds with lower moisture and higher oil content [40]. They also stated that varietal differences that influence seed characteristics (moisture and oil content, hull and testa thinness, and pore number and size) could affect oil extraction by modifying seed pressing behavior, oil flow and kernels permeability.

The PEF pretreatments did not increase oil extraction from dry pecan nut kernels, contrasting with previous findings where the application of 0.8 kJ/kg increased OEY_TOTAL_ of fresh kernels up to 74.8% being higher than OEY of kernels without soaking nor PEF pretreatment [18]. Sarkis et al. reported similar results where drying and grinding of sesame seeds yielded higher values than the seeds pretreated by PEF [4]. Nikiforidis stated that soaking of seeds causes cell swelling, changing oleosomes shape and diffusion kinetics [12]. Consequently, the soaking of dry pecan nuts might hinder oil extraction by the initial reduction of oil content caused by oleosomes release and the reorganization of the remaining oleosomes within the cotyledon tissue trapping them within kernels microstructure.

The analysis of oil stability showed that oils extracted from dry pecan nuts were within the acidity range accepted by the Food and Agriculture Organization for cold-pressed oils [41]. Furthermore, the AC of extracted oils was slightly lower than those achieved in the study employing fresh kernels but higher than those reported for pecan nut oil obtained by solvent extraction [18,28]. Regarding OSI values, Oro et al. reported a similar OSI value for pecan nut oil also extracted by mechanical pressing [42]. Phytosterols concentration of oil extracted from reference kernels was lower in comparison with other studies where pecan nut oil was characterized [8,43,44]. Differences in acidity, AC, OSI and phytosterols concentration might be related to varietal differences between pecan nuts as well as tocopherols concentration since these compounds have been associated to the antioxidant capacity of pecan nut oil [40,44]. Concerning the LOX activity of reference kernels, this value was similar to those reported for different cultivars of walnuts [45]. In tree nuts, LOX inactivation has been associated to moisture reduction [11,46]. Thus, it is possible that the increment of LOX activity observed in control kernels could be related to moisture increase by modifying cell and organelle structure. Instead, LOX activity maintenance observed in PEF-treated kernels compared to control kernels might be due to the improvement of condensed tannins which have been reported to inhibit LOX [47,48,49].

Total phenolics, condensed tannins, and antioxidant capacity of cakes generated from dry kernels were lower than those reported for fresh kernels [18]. These differences could be due to the effect of drying as this process has been demonstrated to decrease TP, CT and AC of pecan nuts [50]. Despite that, the application of PEF increased not only the CT concentration but also enhanced AC of the cakes. Considering that pecan nuts AC is closely related to their CT concentration, the increment of AC in cakes from PEF-treated kernels might be attributed to the release of simple phenolic compounds while condensed tannins are retained in the cake increasing its AC [50,51].

## 5. Conclusions

The application of PEF to dry pecan nuts did not increase OEY nor OEY_TOTAL_ of pretreated kernels (69.1–74.3% and 79.8–85.0%, respectively) in comparison with kernels without soaking nor PEF processing (88.7%). These results could be due to changes in oleosomes characteristics and their localization within the cotyledon tissue of dry kernels submitted to water immersion. Kernels processed at 0.8 kJ/kg were used for the microstructural analysis showing a reduction of the cell size and oleosomes fusion in the intracellular space. These changes might help oil flow during mechanical extraction. Comparable values of acidity, AC, OSI and LOX activity between extracted oils showed that the application of PEF did not negatively affect pecan nut oil quality. Furthermore, the 0.8 kJ/kg pretreatment increased CT and AC of generated cakes compared to cakes from untreated kernels and kernels soaked for 3 min. These results showed that the effectiveness of PEF to increase OEY_TOTAL_ of pecan nuts is dependent on not only kernelsʹ moisture and oil content but also on the drying process. It appears that the rehydration of dry kernels produced a negative effect on their microstructure and oleosomes characteristics. Thus, further research focused on these variables is necessary along with other microscopy techniques and analytical procedures to better understand the effect of PEF pretreatments on oil extraction from pecan nuts.

## Figures and Tables

**Table 1:** Acidity and antioxidant capacity (AC) of oils extracted from dry pecan nuts.

	Reference	*W* (kJ/kg)					
		0.8		7.8		15.0	
		Control	PEF	Control	PEF	Control	PEF
Acidity mg KOH/100 g	21.4 ± 1.1	19.1 ± 0.9	19.9 ± 1.0	20.5 ± 0.1	21.8 ± 0.9	19.0 ± 0.9	21.7 ± 1.0 *
AC mg Trolox EQ/100 g	48.6 ± 0.8	45.7 ± 2.2	47.8 ± 1.3	49.0 ± 1.7	48.3 ± 2.8	48.2 ± 1.7	47.1 ± 1.2

*W*, specific energy input. Reference kernels were not soaked nor PEF processed. Control kernels were placed in tap water for 3, 20 and 35 min corresponding to PEF-treated kernels processed at 0.8, 7.8 and 15.0 kJ/kg, respectively. Oil acidity and AC were expressed per 100 g of pecan nut oil. Means with an asterisk indicated a significant difference from the control (α = 0.05).

**Table 2:** Phytosterols concentration of oils extracted from dry pecan nuts.

		Reference	*W* (kJ/kg)	
			0.8	
			Control	PEF
Phytosterols mg/kg	β-sitosterol Stigmasterol	929.0 ± 89.3	858.2 ± 62.6	910.5 ± 132.2
		501.5 ± 79.7	352.1 ± 17.8	324.4 ± 48.5

*W*, specific energy input. Reference kernels were not soaked nor PEF processed. Control kernels were placed in tap water for 3 min corresponding to PEF-treated kernels processed at 0.8 kJ/kg. Concentrations were expressed per kg of pecan nut oil. Means were not significantly different from the control (α = 0.05).

**Table 3:** Phenolic compounds and antioxidant capacity (AC) of cakes generated from dry pecan nuts.

	Reference	*W* (kJ/kg)					
		0.8		7.8		15.0	
		Control	PEF	Control	PEF	Control	PEF
Total phenolics mmol gallic acid EQ/100 g db	20.0 ± 1.0	20.6 ± 1.8	16.3 ±1.0 *	18.3 ± 2.1	17.9 ± 1.2	18.1 ± 0.7	16.5 ± 0.7 *
Condensed tannins mmol catechin EQ/100 g db	15.4 ± 1.0	17.4 ± 1.6	22.1 ± 2.1 *	13.1 ± 1.3	14.5 ± 0.8 *	13.3 ± 0.8	11.9 ± 1.0 *
AC mmol Trolox EQ/100 g db	14.7 ± 1.0	14.4 ± 1.7	17.9 ± 1.5 *	18.5 ± 1.2	19.2 ± 0.6	16.3 ± 0.4	18.1 ± 1.3 *

*W*, specific energy input. Reference kernels were not soaked nor PEF processed. Control kernels were placed in tap water for 3, 20 and 35 min corresponding to PEF-treated kernels processed at 0.8, 7.8 and 15.0 kJ/kg, respectively. Total phenolics, condensed tannins, and AC were expressed per 100 g of defatted cake. Means with an asterisk indicated a significant difference from the control (α = 0.05).

## Data Availability

The authors confirm that the data supporting the findings of this study are available within the article.
